# Notes on *Amanita* section *Validae* in Hainan Island, China

**DOI:** 10.3389/fmicb.2022.1087756

**Published:** 2023-01-20

**Authors:** Ting Huang, Lin-Jie Su, Nian-Kai Zeng, Serena M. L. Lee, Su-See Lee, Bee Kin Thi, Wen-Hao Zhang, Jing Ma, Hong-Yan Huang, Shuai Jiang, Li-Ping Tang

**Affiliations:** ^1^School of Pharmaceutical Sciences and Yunnan Key Laboratory of Pharmacology for Natural Products, Kunming Medical University, Kunming, China; ^2^College of Pharmacy-Transgenic Laboratory, Hainan Medical University, Haikou, China; ^3^National Parks Board, Singapore, Singapore; ^4^Forest Research Institute Malaysia, Kepong, Selangor, Malaysia

**Keywords:** Amanitaceae, phylogeny, taxonomy, tropics, biodiversity, macrofungi, mushrooms

## Abstract

Hainan is the second largest island in China with the most extensive and well-preserved tropical forests and is also the largest island of the Indo Burma Biodiversity Hotspot. It provides *in situ* conservation for the unique ecosystem of the island. Recent studies have shown that there are diverse fungal species in Hainan. In this study, about 40 collections of the genus *Amanita* have been studied based on the morphology and molecular systematics, including 35 Chinese specimens (24 from Hainan, and eleven from other regions) and three specimens from other countries (Singapore and Malaysia). In total, five new species belonging to *Amanita* section *Validae* are described: *A. cacaina*, *A. parvigrisea*, *A. pseudofritillaria*, *A. pseudosculpta*, and *A. yangii. Amanita parvifritillaria* is recorded for the first time in Hainan. It is also the first report of this fungus occurring, outside Yunnan Province, China. Among the five new species, two are unique in this section because of the appendiculate pileus margin and the absence of an annulus. Based on these new findings, the diagnosis of the section *Validae* should be slightly modified to include a few species with appendiculate margin and the lack of annulus.

## Introduction

Hainan, with an area of about 33,900 km^2^, is located in the south China Sea and the southernmost of China. It is the second largest island in China and the largest island of the Indo Burma Biodiversity Hotspot. With a network of 54 terrestrial protected areas, Hainan has the most extensive and the best-preserved tropical forests, which provide *in situ* conservation for the unique ecosystem of the island. The island is famous for its rich biological resources and has at least 397 endemic plant species (Francisco-Ortega et al., 2010; Lu et al., 2011; Jiang et al., 2013; Chen, 2014). Recent publications also have shown that the island has a high diversity of fungi (Zeng et al., 2014; An et al., 2017; Wu et al., 2019; Jiang et al., 2021).

*Amanita* Pers., with more than 700 species, is widely distributed around the world.^1^ Based on the morphology, phylogeny, and basidiomata lifestyle, this genus is divided into three subgenera (*Amanita*, *Amanitina*, and *Lepidella*) and eleven sections. Namely, subgenus *Amanita* Pers. consists of four sections: (1) sect. *Amanita* Pers., (2) sect. *Amarrendiae* (Bougher & Lebel) Zhu L. Yang, Yang-Yang Cui, Qing Cai & Li-Ping Tang, (3) sect. *Caesareae* Singer ex Singer, and (4) sect. *Vaginatae* (Fr.) Quél. Subgenus *Amanitina* (E.J. Gilbert) E.J. Gilbert contains six sections: (1) sect. *Amidella* (J. E. Gilbert) Konrad & Maubl., (2) sect. *Arenariae* Zhu L. Yang, Yang-Yang Cui & Qing Cai, (3) sect. *Phalloideae* (Fr.) Quél., (4) sect. *Roanokenses* Singer ex Singer, (5) sect. *Strobiliformis* Singer ex Zhu L. Yang, Yang-Yang Cui & Qing Cai, and (6) sect. *Validae* (Fr.) Quél.; and subgenus *Lepidella* Beauseigneur includes only one section, *viz*. sect. *Lepidella* Corner & Bas (Cui et al., 2018).

Section *Validae* is recognized by a pileal margin that is non-striate and non-appendiculate; stipe base with globose to subglobose or marginate; annulus membranous, dominantly composed of filamentous hyphae; volval remnants often as verrucae, warts, flocci, or patches, occasionally as short limb; basidiospores amyloid; and clamps absent (Corner and Bas, 1962; Yang, 2005, 2015; Cui et al., 2018).

In China, 166 species of *Amanita* have been reported, including 20 taxa belonging to the sect. *Validae* (Yang et al., 2001; Yang, 2005, 2015; Cui et al., 2018, 2022; Zhang et al., 2020, 2021; Mu et al., 2021; Zhong et al., 2021). Currently, 27 species of *Amanita* have been reported in Hainan. Among them, three species belong to the sect. *Validae*: *viz. A. innatifibrilla* Zhu L. Yang ex Zhu L. Yang, Yang-Yang Cui, & Qing Cai, which was originally described from Hainan, *A. fritillaria* Sacc. and *A. sinocitrina* Zhu L. Yang, Zuo H. Chen, and Z. G. Zhang, which were originally described from India and Hunan Province of China, respectively (Yang, 2005; Cui et al., 2018; Zhang et al., 2020).

In the last 5 years, we have been collecting fungi from the protected regions of Hainan Island. In this study, we present new findings on *A.* sect. *Validae*. Based on the morphological examination and phylogenetical analyses, five new species are described and one new record for Hainan is reported. The species concept of the section *Validae* is modified according to our new data. Other results will be published in the future.

## Materials and methods

### Collection sites

Hainan has a tropical climate, which is dominated by the summer monsoon with a rainy season from May to October and a dry season extending from November to April. The eastern part of the island receives annual precipitation of 2,000-2,400 mm while the western part has 1,000-2,000 mm of rainfall with an average of over 1,600 mm (Hsieh and Zhong, 1990). All the collections came from two reserve areas: Jianfenling and Yinggeling. Both are located in the southern part of the island. Jianfenling, which was established in 1960, is the oldest forest reserve in Hainan, while Yinggeling, which was established in 2003, is the largest primary rainforest in Hainan. The vegetation in these two areas consists of tropical monsoon forests with evergreen, deciduous elements, and some small patches of tropical bamboo forests at altitudes ranging from 350 to 1,400 m above sea level (a.s.l). Elfin and tropical evergreen forests are found at higher elevations (above 1,300 m a.s.l) and are dominated by lichens, mosses, small trees/shrubs, and conifers (*Keteleeria* spp., *Nageia* spp., *Pinus* spp., and *Podocarpus* spp.). The specimens in this study were collected opportunistically from 2015 to 2020 from Jianfenling and Yinggeling. Fresh basidiomata were photographed in the field and field data including habitats and locations were recorded. We also researched some collections from other regions of China (Guizhou and Yunnan Provinces) and other countries (Malaysia and Singapore). The specimens were deposited in the Fungal Herbarium of Hainan Medical University (FHMU), Forest Research Institute Malaysia (FRIM), the Mycological Herbarium of Kunming Medical University (MHKMU), and the National Parks Board Singapore (SING).

### Morphology

Macroscopic descriptions of the basidiomata were based on field notes and digital photographs. The color was described using the color codes from Kornerup and Wanscher (1981). Microscopic characters were described from the dried specimens after being sectioned, mounted in 5% KOH, and observed under the light microscope. Congo red was used when necessary and Melzer’s reagent was used to examine the amyloid of basidiospores. The number of measured basidiospores was expressed as [n/m/p], which represents n basidiospores from m basidiomata of p collections. Dimensions for basidiospores were given using (a) b–c (d), where the range of “b–c” indicates a minimum of 90% of the measured values, and “a” and “d” are extreme values. Q refers to the length/width ratio in the side view of basidiospores; and Q_*m*_ indicates the average Q of measured basidiospores ± sample standard deviation.

### DNA extraction, amplification, and sequencing

Total genomic DNAs were extracted from 10 to 20 mg of dried basidiomata using a modified cetyltrimethylammonium bromide method (CTAB) (Doyle and Doyle, 1987). In total, three DNA gene fragments, the nuclear ribosomal DNA Internal transcribed space (ITS) regions, large subunit nuclear ribosomal RNA (nrLSU), and the second largest subunit of RNA polymerase II (*rpb2*), were amplified by polymerase chain reaction (PCR) using the primer pairs ITS5/ITS4, LR0R/LR5, and *rpb2*-6F/*rpb2*-7R, respectively (Vilgalys and Hester, 1990; White et al., 1990; Cai et al., 2014). Procedures for total DNA extraction and PCR condition followed the references therein (Tang et al., 2015, 2017). Amplified PCR products were sequenced using an ABI 3730 DNA Analyzer (Sangon, Shanghai, China) with the same primers.

### Sequence alignment and phylogenetic analyses

*Amanita* sect. *Validae* is sister to sect. *Strobiliformis* as reported by Cui et al. (2018). Thus, three representatives from the sect. *Strobiliformis*, *A. aspericeps* Yang-Yang Cui, Qing Cai and Zhu L. Yang, *A. griseoverrucosa* Zhu L. Yang, and *A. strobiliformis* (Paul. ex. Vitt.) Bertillon were chosen as an outgroup in the concatenated matrix. In the ITS phylogeny, outgroups were excluded to ensure the reliability of alignment, as the exclusion of outgroups can reduce the gaps in the dataset. The nrLSU and ITS datasets include all available sequences of sect. *Validae* from GenBank.

DNA sequences were assembled with SeqMan (DNASTAR Lasergene 9), aligned using MUSCLE v3.6 (Edgar, 2004), and manually adjusted where necessary in BioEdit 7.0.9 (Hall, 1999). The datasets were analyzed with maximum likelihood (ML) and Bayesian inference (BI). ML analyses with 1,000 rapid bootstrap replicates were performed in RAxML 7.0.3 (Stamatakis et al., 2008); GTRGAMMA was set as the default model; statistical support of clades was obtained with 1,000 rapid bootstrap replicates. The best-fit model of nucleotide substitution was obtained in MrModeltest 2.3 (Nylander, 2004). The selected models (invgamma) were the same for ITS and the concatenated database. Overall, four simultaneous Markov chains were run for 1,000 generations and sampled every 1,000 generations. At the end of the run, the average deviation of split frequencies was lower than 0.01 (Ronquist et al., 2012).

## Results

In total, 55 sequences (23 ITS, 22 nrLSU, and 10 *rpb2*) were newly generated in this study. Other sequences were downloaded from GenBank.^2^ All sequences with their relevant information used in this study are listed in Table 1. The alignment is available at TreeBase (accession nos. 29763 and 29764). The ITS matrix contains 58 sequences representing 41 taxa (without outgroups), and the alignment has 1,110 nucleotide sites. The concatenated matrix (nrLSU-*rpb2*) contains 40 sequences representing 35 taxa (including five outgroups), and the alignment has 3,901 nucleotide sites.

**TABLE 1:** Information of sequences used in this study.

Taxon	Voucher	Locality	Genebank acession number		
			**LSU**	**ITS**	**RPB2**
*A. orsonii*	HKAS 52264	Yunnan, China	MH486714	MH508474	MH486145
*Amanita ahmadii*	SJ35	Pakistan	KY996725	KY996724	–
*A. arenaria*	VPI679	Australia	GQ925382	–	–
*A. arenaria*	VPI363	Australia	GQ925384	–	–
*A. aspericeps*	HKAS100519	Fujian, China	MH486369	MH508255	MH485864
*A. augusta*	HKAS101418	USA	MH486375	–	MH485869
*A. brunneolimbata*	HKAS 101392	Guangdong, China	MH486398	MH508272	MH485889
*A. brunneolocularis*	ANDES_F313 NVE57	Colombia	FJ890044	FJ890033	–
*A. brunnescens*	AFTOL-ID 673	USA	AY631902	–	AY780936
*A. brunnescens*	RET 529-10	USA	KP284284	KP284273	–
*A. cacaina*	MHKMU NK Zeng 2509	Hainan, China	ON768726	ON768706	–
*A. cacaina*	MHKMU NK Zeng 2557	Hainan, China	ON768725	ON768705	–
*A. cacaina*	MHKMU NK Zeng 3064	Hainan, China	ON768727	–	–
*A. cacaina*	MHKMU NK Zeng 3192	Hainan, China	ON768728	ON768707	–
***A. cacaina***	**MHKMU NK Zeng 5027 (T)**	**Hainan, China**	**ON768734**	**ON768710**	–
*A. castanea*	MFLU 15-1424	Thailand	KU877539	KU904823	–
*A. citrina var. grisea*	LEM9 70501	Japan	–	AB015680	–
*A. citrinoannulata*	HKAS 83459	Chongqing, China	MH486464	MH508318	MH485944
*A. citrinoindusiata*	HKAS 100522	Yunnan, China	MH486468	MH508320	MH485947
*A. congolensis*	RET 346-6	Zambia	HQ539736	KR919753	–
*A. detersa*	HKAS 71476	Yunnan, China	MH486475	MH508328	MH485954
*A. echinulata*	KM 87	DBR, Cameroon	MT446291	MT446259	
*A. elongata*	RET 384-5	Canada	MH486489	MH508337	MH485967
*A. flavipes*	HKAS56824	Yunnan, China	MH486500	MH508342	MH485974
*A. flavoconia*	RET 439-8	CT, USA	MH486511	MH508348	MH485983
*A. flavorubescens*	F:PRL 6062	USA	–	GQ166902	–
*A. flavosquamosa*	HKAS 83692	Yunnan, China	MH486521	MH508356	MH485991
*A. franchetii*	DBBJUS01	Spain	–	JX515563	–
*A. fritillaria*	HKAS100520	Fujian, China	MH486527	MH508359	MH485995
*A. fritillaria*	MHKMU LP Tang3253	Hainan, China	ON768729	ON768712	
*A. fritillaria*	MHKMU M Mu691	Hainan, China	ON768739	ON768718	OP056469
*A. fritillaria*	MHKMU NK Zeng4437	Hainan, China	–	ON768711	–
*A. fritillaria*	MHKMU T Huang406	Hainan, China	ON768737	ON768715	–
*A. fritillaria*	MHKMU T Huang410	Hainan, China	ON768738	ON768709	–
*A. fuscosquamosa*	PDD92862	New Zealand	MH486558	MH508373	–
*A. griseoverrucosa*	HKAS 100613	Anhui, China	MH486579	MH508390	MH486041
*A. guyanensis*	TH 9767	Guyana	MK105502	MK064192	MK092929
*A. intermedia*	MCVE-30172	Italy	MN257622	MN257615	MN267406
*A. lacerosquamosa*	Li 150829-44	Yunnan, China	MN647043	MN647014	MN657456
*A. morrisii*	RET 271-7	USA	KT213442	KT213441	–
*A. novinupta*	RET 060-2	USA	KF561978	KF561974	–
*A. orsonii*	RET 717-8	India	KX270345	KX270327	–
*A. parvifritillaria*	MHKMU NK Zeng 3596	Yunnan, China	ON768721	ON768701	
*A. parvifritillaria*	HKAS 83737	Yunnan, China	MH486749	MH508494	MH486173
*A. parvifritillaria*	MHKMU T Huang 412	Hainan, China	ON768733	ON768714	OP056474
*A. parvigrisea*	MHKMU NK Zeng 2538	Hainan, China	ON768724	ON768704	–
***A. parvigrisea***	**MHKMU LP Tang 3251 (T)**	**Hainan, China**	**ON768741**	**ON768716**	–
*A. porphyria*	MB-100156 (duplicate HKAS 84871)	Germany	MH486762	MH508507	MH486181
*A. pseudofritillaria*	MHKMU NK Zeng 3051	Hainan, China	ON768722	ON768702	
*A. pseudofritillaria*	MHKMU NK Zeng 4268	Hainan, China	ON768736	–	OP056470
*A. pseudofritillaria*	MHKMU NK Zeng 3433	Hainan, China	ON768723	ON768703	–
***A. pseudofritillaria***	**MHKMU T Huang 398 (T)**	**Hainan, China**	–	**ON768708**	**OP056467**
*A. pseudofritillaria*	MHKMU T Huang 408	Hainan, China	–	ON768717	OP056468
*A. pseudofritillaria*	MHKMU NK Zeng 4270	Hainan, China	–	ON768713	–
***A. pseudosculpta***	**MHKMU LP Tang 3167 (T)**	**Hainan, China**	**ON768735**	–	**OP056472**
*A. pseudosculpta*	MHKMU T Huang 342	Hainan, China	ON768740	OP106386	OP056471
*A. pseudosculpta*	MHKMU WH Zhang 379	Hainan, China	ON768730	–	–
*A. rubescens*	HKAS 100525	Shandong, China	MH486808	MH508552	MH486220
*A. rubescens*	HKAS 101398	France	MH486811	–	MH486222
*A. sculpta*	SL1560_A09	Singapore	ON768742	ON768719	–
*A. sculpta*	SL1560_A10	Singapore	–	ON768720	–
*A. sepiacea*	HKAS 100604	Anhui, China	MH486845	MH508582	MH486254
*A. silvicola*	RET 594-9	USA	–	KR919766	
*A. sinocitrina*	HKAS 100530	Guizhou, China	MH486873	MH508598	MH486279
*A. solaniolens*	Smith-2018 iNaturalist # 17338999	USA	–	MK573911	–
*A. spissa*	HKAS 92051	Huanren, Liaoning, China	MH486892	MH508611	MH486295
*A. spissa*	HKAS 100533	France	MH486891	–	MH486294
*A. spissacea*	HKAS 71041	Hokkaido, Japan	MH486888	MH508610	MH486292
*A. strobiliformis*	MB-001177 (duplicate HKAS84872)	Germany	MH486895	MH508614	MH486298
*A. westii*	BM SH26	USA	HQ539759	–	–
***A. yangii***	**MHKMU M Mu 660 (T)**	**Hainan, China**	**ON768731**	**ON768743**	**OP056473**
*A. yangii*	MHKMU X Na 379	Hainan, China	ON768732	–	–

T means the holotype collection and the information of the holotype in bold.

**FIGURE 1:**
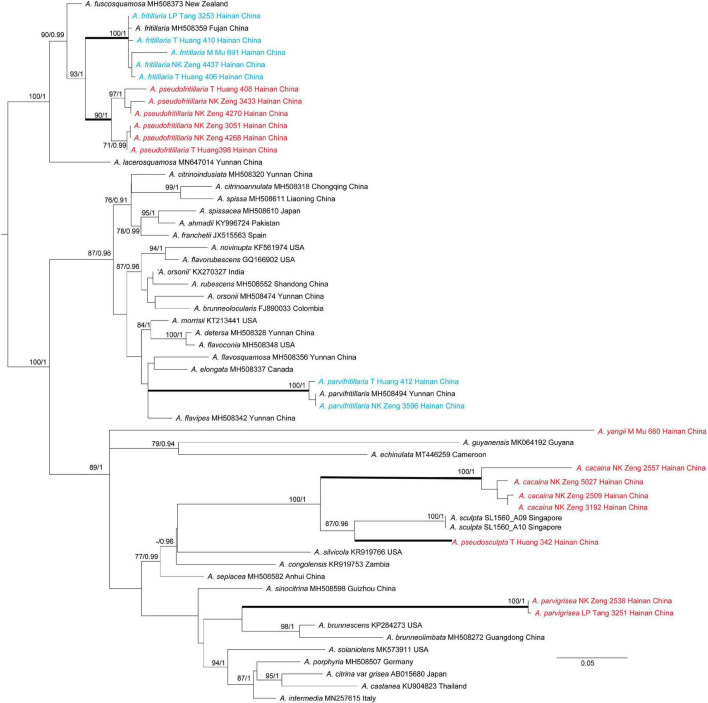
Phylogenetic tree of *Amanita* sect. *Validae* based on ITS: RAxML BP values (≥ 70%) are shown above branches, Bayesian posterior probabilities (≥ 0.90) are shown above branches. New species are highlighted in red and new record species in blue.

**FIGURE 2:**
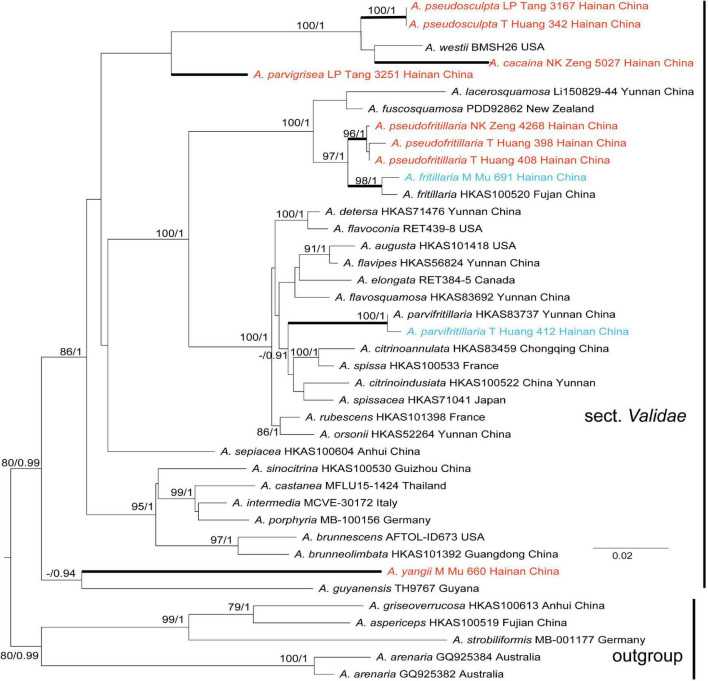
Phylogenetic tree of *Amanita* sect. *Validae* based on two-gene dataset (nrLSU and *rpb2*): RAxML BP values (≥ 70%) are shown above branches, Bayesian posterior probabilities (≥0.90) are shown above branches. New species are highlighted in red and new record species in blue.

Tree topologies derived by ML and BI analyses from the same datasets were almost identical, while statistical support showed slight differences, but the two species had no *rpb2* sequence (Figures 1, 2). In different phylogenetic trees (ITS, and the concatenated tree), 24 specimens from Hainan formed seven lineages representing seven distinct species, *viz.* five new species (*A. cacaina*, *A. parvigrisea*, *A. pseudofritillaria*, *A. pseudosculpta*, and *A. yangii*) and two known taxa (*A. fritillaria* and *A. parvifritillaria* Y.Y. Cui, Q. Cai & Zhu L. Yang). Among them, the sister relationships among some species were resolved while the other species had no solid sister. *Amanita cacaina* clustered together with *A. pseudosculpta* and *A. westii* (Murrill) Murrill with strong support, but their relationship needs further study. *Amanita pseudofritillaria* grouped with *A. fritillaria* with 100% BP value in our study (Figure 2), but the latter was sister to *A. fuscosquamosa* A. E. Wood in Cui et al. (2018). In the concatenated tree, *A. yangii* clustered with *A. guyanensis* Mighell and T.W. Henkel with moderate support (BP 83%). The sister relationship between *A. pseudosculpta* and *A. sculpta* Corner and Bas was strongly supported by ITS data. *Amanita parvigrisea* grouped with *A. echinulata* Beeli and *A. guyanensis* with weak support in ITS while clustered with *A. cacaina, A. pseudosculpta*, and *A. westii* with 69% BP value in the concatenated tree. The sister relationship of *A. parvifritillaria* has not been resolved in this study and the previous multi-locus phylogenetic analysis (Cui et al., 2018).

### Taxonomy

*Amanita cacaina* L.P. Tang, T. Huang & N.K. Zeng, sp. nov.

**FIGURE 3:**
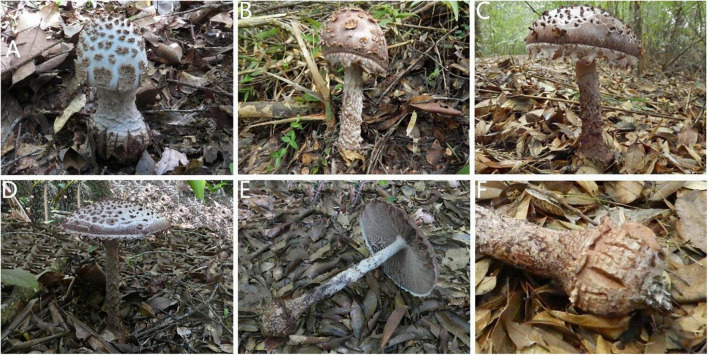
Basidiomata of *Amanita cacaina*: **(A)** from MHKMU N.K. Zeng 3064; **(B)** from MHKMU N.K. Zeng 2509; **(C,F)** from MHKMU N.K. Zeng 2557; **(D,E)** from MHKMU N.K. Zeng 5027, holotype.

**FIGURE 4:**
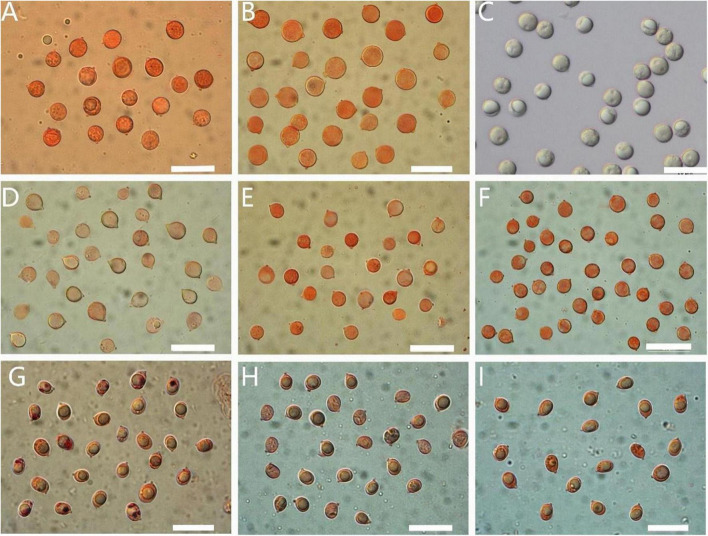
Basidiospores of *Amanita* sect. *Validae*: **(A)** *A. cacaina* from MHKMU N.K. Zeng 5027; **(B)** *A. pseudosculpta* from MHKMU L.P. Tang 3167; **(C)** *A. sculpta* from SL1560; **(D)** *A. parvigrisea* from MHKMU L.P. Tang 3251; **(E)** *A. parvifritillaria* from MHKMU T. Huang 412; **(F)** *A. yangii* from MHKMU M. Mu 660; **(G)** *A. fritillaria* from MHKMU T. Huang 410; **(H)** *A. pseudofritillaria* from MHKMU T. Huang 398; **(I)** *A. pseudofritillaria* from MHKMU N.K. Zeng 4270. *Bars*: 20 μm.

**FIGURE 5:**
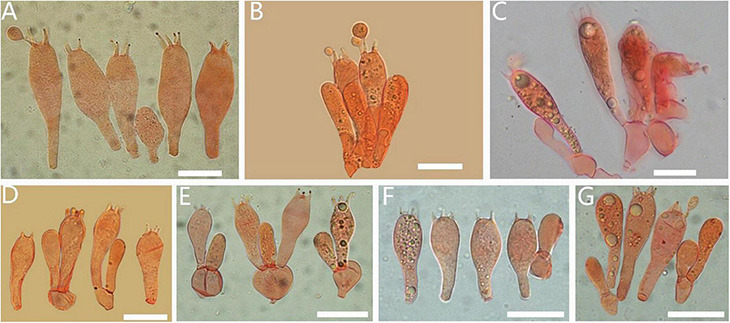
Basidia of *Amanita* sect. *Validae*: **(A)** *A. cacaina* (MHKMU N.K. Zeng 5027); **(B)** *A. pseudosculpta* (MHKMU L.P. Tang 3167); **(C)** *A. sculpta* (SL1560); **(D)** *A. parvigrisea* (MHKMU L.P. Tang 3251); **(E)** *A. parvifritillaria* (MHKMU T. Huang 412); **(F)** *A. pseudofritillaria* (MHKMU T. Huang 398) **(G)** *A. yangii* (MHKMU M. Mu 660). *Bars*: 20 μm.

**FIGURE 6:**
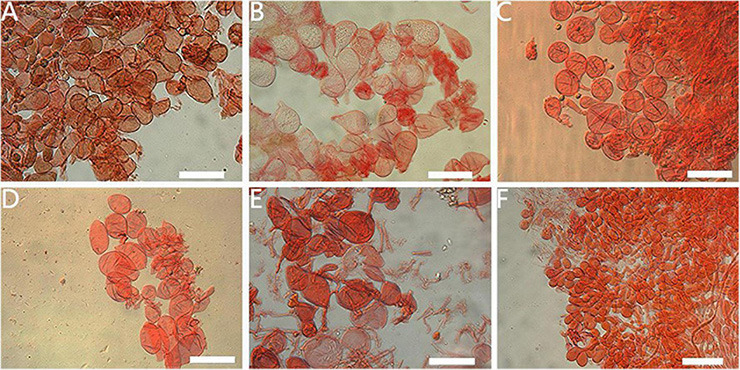
Lamella edge of *Amanita* sect. *Validae*: **(A)** *A. cacaina* (MHKMU N.K. Zeng 5027); **(B)** *A. pseudosculpta* (MHKMU L.P. Tang 3167); **(C)** *A. parvigrisea* (MHKMU L.P. Tang 3251); **(D)** *A. parvifritillaria* (MHKMU T. Huang 412); **(E)** *A. pseudofritillaria* (MHKMU T. Huang 398); **(F)** *A. yangii* (MHKMU M. Mu 660). *Bars*: 50 μm.

**FIGURE 7:**
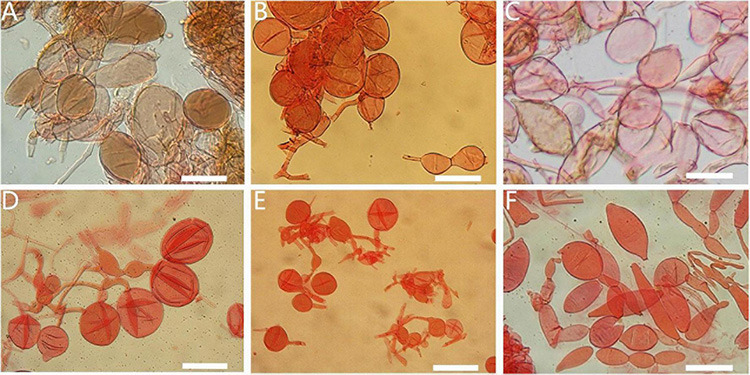
Veil remnants on plieus of *Amanita* sect. *Validae*: **(A)** *A. cacaina* (MHKMU N.K. Zeng 5027); **(B)** *A. pseudosculpta* (MHKMU L.P. Tang 3167); **(C)** *A. sculpta* (SL1560); **(D)** *A. parvifritillaria* (MHKMU T. Huang 412); **(E)** *A. pseudofritillaria* (MHKMU T. Huang 398); **(F)** *A. yangii* (MHKMU M. Mu 660). *Bars*: 50 μm.

**FIGURE 8:**
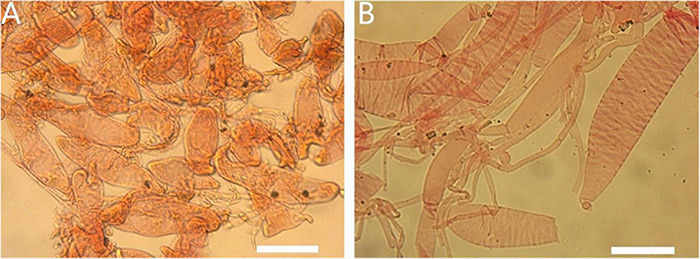
Appendiculate of plieus margin in the *Amanita* sect. *Validae*: **(A)** *A. cacaina* (MHKMU N.K. Zeng 5027); **(B)** *A. pseudosculpta* (MHKMU L.P. Tang 3167). *Bars*: 50 μm.

MycoBank no.: MB 844369 (Figures 3, 4A, 5A, 6A, 7A, 8A).

Holotype—China. Hainan Province, Baisha Li Autonomous County, Yinggeling Nature Reserve, 19°1′19″N, 109°23′48″E, elevation 550 m, 4 September 2020, *N.K. Zeng 5027* (MHKMU N.K. Zeng 5027); GenBank accessions: ON768734 (ITS), ON768734 (LSU).

Etymology—*cacaina*, from *cacaina* = cacao, refers to the color of basidiomata like cacao or chocolate brown when aged or bruised.

Diagnosis—similar to *A. sculpta* but differs in its smaller basidiomata, whitish pileus at bud stage, pyramidal to angular warts on pileus, usually felted into patches at young, and globose basal bulb adorned with small warts.

*Basidiomata* very large. *Pileus* up to 18 cm in diam., at first hemispheric, then convex to applanate, slightly exceeding gills, white to dingy white (1A1) when young, finally the whole pilei surface turning cacao brown, dark brown or chocolate brown (6D5, 6E4) when age or bruising; volval remnants on pileus warts up to 0.5 cm high at the center, gradually diminishing in size toward the margin of pileus, pyramidal to angular, often lumpish or felted into patches at young, dingy white to grayish white (2B1–2) at bud period, dark brown to chocolate brown (6D5, 6E4) when mature or bruising, easily breaking off or washed away by rain; margin non-striate, appendiculate with ragged, floccose or cottony, remnants of the partial veil, dingy white (1A1) when young, brownish (6D3) when mature or bruising; *context* 1–1.5 cm thick at the center, white to gray white (1A1) at young, turning brownish to linoleum brown (5E4–8) when cutting or bruising. *Lamellae* 1–1.3 cm in width, nearly free, rather crowded, 220–240 pieces of primaries with 1–2 shorter ones between each pair, white (1A1) when young, chocolate-brown to dark brown (6E4, 4E4–6) when mature, often with a minutely floccose denticulate edge; lamellae attenuate. *Stipe* 23–25 cm long, 2.2–2.7 cm in diam., more or less thickened downward, surface covered with dirty white to whitish (1A1, 2B1) floccose to cottony remnants of the partial veil, camel brown to brownish (6D3) even to blackish (18D1, 18E1) when bruising, sometimes with rim or brownish small warts at bulb base; *basal bulb* 5–5.2 cm in diam., globose to subglobose, adorned with small brownish squarish warts, forming several rims; *context* same as the context of the pileus, whitish at young, brown when bruising. *Annulus* absent, poorly developed in shape even at bud period, floccose to cottony, friable, dingy white to brownish, leaving at the edge of the pileus, at edges of lamellae, and on the stipe. All parts of young primordium are white to dingy white, but taking on the pale chocolate or cacao powder hue of the whole fruit body including flesh at mature. *Taste and Odour* no records found.

*Basidiospores* [60/2/2] (8.0) 8.5–10.0 (11.0) × 8.0–10.0 (10.5) μm, Q = 1–1.06 (1.09), Q_*m*_ = 1.02 ± 0.13, mostly globose, sometimes subglobose, colorless, thin-walled, smooth, amyloid, apiculus small. *Basidia* 35–60 × 13–19 μm, clavate, 4-spored; sterigmata straight, 4–7 μm long; basal septa lacking clamps. *Lamellar trama* bilateral. Mediostratum 15–50 μm wide, made up of abundant inflated cells 50–100 × 16–29 μm, subfusiform, ellipsoid to clavate; mixed with filamentous hyphae 2–7 μm in diam.; vascular hyphae scarce. The lateral stratum is made up of abundant inflated cells 56–125 × 11–30 μm, subfusiform to ellipsoid; mixed with filamentous hyphae 3–10 μm diam. Subhymenium 25–45 μm thick, with 2–3 layers of cells 12–22 × 10–21 μm, subglobose to ellipsoid or irregular. *Lamellar edge* somewhat gelatinized, dominately composed of inflated cells 25–50 × 15–20 μm, hyaline, ellipsoid, clavate or pyriform, single or in short chains; mixed with scattered filamentous hyphae 2–7 μm diam. *Marginal cells* abundant, forming a thick sterile margin along edges of gills, 40–120 × 16–40 μm, subfusiform to fusiform, terminal on filamentous hyphae 3–10 μm diam. *Volva remnants (warts) on pileus* composed of vertically to irregularly arranged elements: inflated cells very abundant, 20–90 × 12–67 μm, subglobose, subfusiform to ellipsoid, colorless to yellowish, thin-walled, terminal or in chains of 2–3; filamentous hyphae rare to locally abundant, 2–12 μm in diam., colorless or yellowish, thin-walled, branching; vascular hyphae scarce. *Volva remnants on stipe* are similar to the structure of volva remnants (warts) on pileus, but with more abundant filamentous hyphae and longer inflated cells. *Volva remnants on stipe base* composed of abundant to dominated filamentous hyphae, 2–12 μm in diam., colorless or yellowish, thin-walled, branching; mixed with scarce inflated cells 20–80 × 22–73 μm, subglobose, subfusiform to ellipsoid, colorless to yellowish, thin-walled, terminal or in chains of 2–3; vascular hyphae scarce. *Pileipellis* 60–100 μm thick; upper layer 32–50 μm thick, non- or slightly gelatinized, composed of subradially to somewhat interwoven filamentous hyphae 2–12 μm in diam., thin-walled, colorless or yellowish; lower layer 20–65 μm thick, composed of radially and compactly arranged filamentous hyphae 2–11 μm in diam., colorless or yellowish; vascular hyphae scarce. *Stipe trama* composed of longitudinally arranged terminal cells 60–220 × 19–32 μm, clavate to long clavate; mixed with scattered to abundant filamentous hyphae 3–12 μm in diam.; vascular hyphae scarce. *Clamps* are absent in all parts of basidiomata.

Habitat—Solitary or scattered on soil in a tropical broad-leaved forest; in summer.

Distribution—Hainan Province, China.

Additional specimens examined of *A. cacaina*—China. Hainan Province, Baisha Li Autonomous County, Yinggeling Nature Reserve, 19°1′19″N, 109°23′48″E, elevation 800 m, 3 August 2015, *N.K. Zeng 2509* (MHKMU N.K. Zeng 2509); *ibid.*, elevation 850 m, 5 August 2015, *N.K. Zeng 2557* (MHKMU N.K. Zeng 2557); *ibid.*, elevation 550 m, 4 June 2017, *N.K. Zeng 3064* (MHKMU N.K. Zeng 3064); *ibid.*, elevation 550 m, 30 July 2017, *N.K. Zeng 3192* (MHKMU N.K. Zeng 3192).

Specimens examined of *A. sculpta*—Malaysia. Negeri Sembilan State: Ulu Bendul Recreation Park, elevation 135 m, 21 April 2010, S.S. Lee et al. (no collection number) (FRIM 72469); Pahang State: Pine Tree Trail, Fraser’s Hill, elevation about 1,300 m, 09 Dec 2011, S.S. Lee et al. (no collection number) (FRIM 72557). Singapore. Bukit Timah Nature Reserve, elevation about 70 m, 30 August 2020, Loo et al. SL1560 (SING 0212684).

Notes—*Amanita cacaina* is characterized by a large basidioma (pileus up to 18 cm wide), a white pileus (at bud stage) with whitish pyramidal warts usually felted into patches at young which are easy to be dislodged, the margin of the pileus hanging on whitish to pale chocolate veil remnants, a dark stipe covered with whitish floccose to cottony volval remnants, a globose basal bulb decorated with small warts, and globose basidiospores.

There are several taxa similar to the new one whereby these taxa share some common characteristics in morphological appearance: large and dark basidiomata at mature, conspicuous brown to dark brown warts on pileus, the margin adorned with whitish to brown veil remnants, globose to napiform bulb on stipe base, a strong discoloration of the fruit body, absent clamps, and the tropical or subtropical distribution. However, they can be distinguished by the color of fruit bodies, the shape and size of their warts on pileus, stipe bulbs, basidiospores, and so on.

**FIGURE 9:**
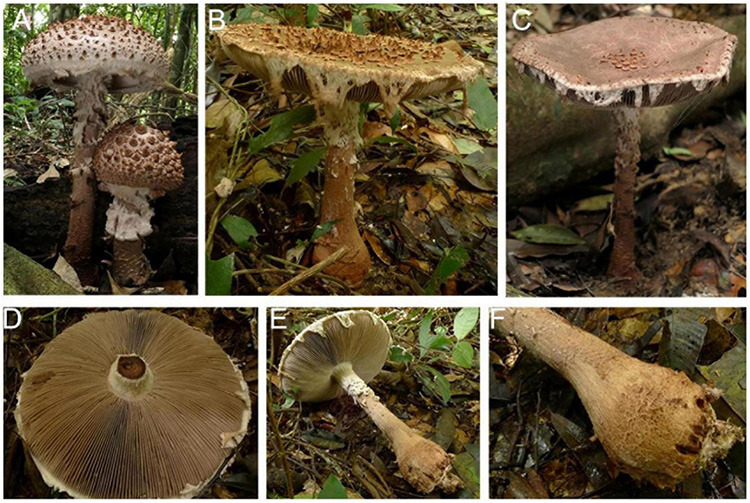
Basidiomata of *Amanita pseudosculpta*: **(A)** from MHKMU T. Huang 342; **(B,D–F)** from MHKMU L.P. Tang 3167, holotype; **(C)** from MHKMU W.H. Zhang 379.

*Amanita pseudosculpta*, a sympatric new taxon to *A. cacaina*, differs in yellow brown or pale brown basidiomata rather than white ones at the bud stage; larger and striking warts (up to 1 cm in height, 0.8 cm in width) on pileus (the largest ones on the center), which come off easily; stipe bulb covered floccose to cottony rather than rim or small warts, fresh dark brown at mature (Figure 9), slightly larger and rounder basidiospores, lamellar edge composed of pyriform inflated cells, discoloration when age or injured, volval remnants on pileus and stipes composed of abundant inflated cells, with a few filamentous hyphae.

**FIGURE 10:**
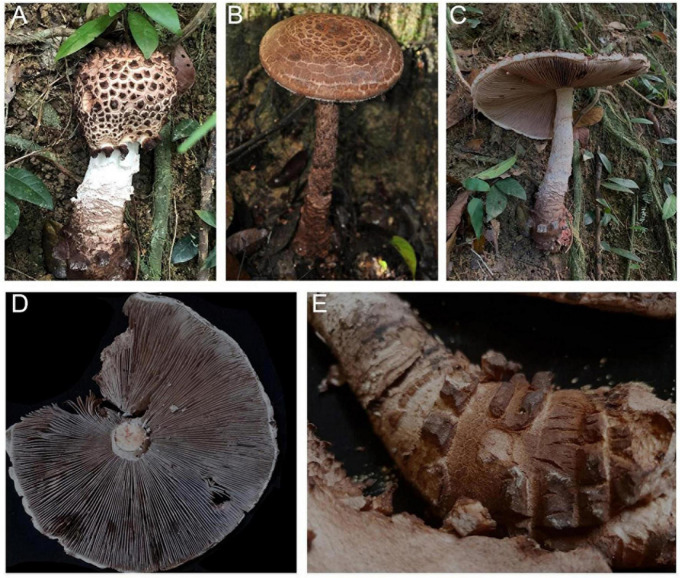
Basidiomata of *Amanita sculpta*: **(A,C–E)** from SL1560; **(B)** from FRIM 72469.

*Amanita sculpta* (Figures 4C, 5C, 7C, 10) originally from Singapore is distinct in fairly large basidiocarps (pileus up to 27 cm wide, stipe up to 26 cm long); pileus dark brown or madder brown at the center, becoming pale ox-blood red, even lighter toward the edges, margin adorned with reddish-brown veil remnants; larger, irregular to pyramidal warts (up to 1.6 cm high, 1.5 cm wide) on pileus (the largest ones on the margin) that washed off by nights rain; stipe with fusiform or napiform bulb, decorated rim or large warts at stipe base; flesh pinkish brown or pale umber; the strong purple to vinaceous brownish discoloration of the whole fruit body; and smell like ripe pear (Corner and Bas, 1962; Bas, 1969; Lee et al., 2021; personal observation of two co-authors, Figure 10). Among these characters, large madder brown warts, fusiform or napiform bulb, and stipe base with rim or large warts are the most obvious diagnostic criteria. It is worth noting that this species was subsequently reported from Asian countries, e.g., China, Japan, and Thailand (Yang, 2005, 2015; Sanmee et al., 2008; Tsujino et al., 2009; Lee, 2017). In the past, *A. cacaina* and *A. pseudosculpta* were likely to be mistaken for *A. sculpta* in China (Fujian, Guangdong, Guangxi, Hainan, Hunan, and Yunnan Provinces) (Yang, 1997, 2005, 2015; Tang, 2015; Cui et al., 2018; Zeng and Jiang, 2020).

*Amanita westii*, a rare species which distributes in Mississippi, Texas, and including the holotype location Florida in the USA, has comparatively smaller basidiomata (pileus up to 15.5 cm wide), small brown pyramidal to somewhat flattened warts (0.2–0.3 cm high) that are not dislodged easily, a prominent obovoid-napiform bulb on the stipe base, flesh that bruise quickly reddish brown, larger and oblong basidiospores (11–12.8 × 6.6–7.3 μm, Q_*m*_ = 1.73), a slight odor of anise, and the color of wine-red on dried specimens (Murrill, 1944; Bas, 1969; Tulloss and Lewis, 1994; Kuo, 2017).

The four above-mentioned taxa show a close relationship in morphology, especially the spores. Of these, three Asian taxa have similar sizes and shapes of spores. In Cui et al. (2018), *A. sculpta* was placed into the section *Roanokenses*, but no molecular evidence is provided while there was no sequence available in public databases. Our molecular data first confirm that the species from Singapore, including *A. westii* and another two new taxa (*A. cacaina* and *A. pseudosculpta*), are members of the section *Validae*. However, the sister relationship of *A. cacaina* cannot be resolved according to the present data.

*Amanita parvigrisea* L.P. Tang, T. Huang & N.K. Zeng, sp. nov.

**FIGURE 11:**
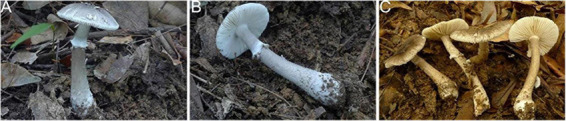
Basidiomata of *Amanita parvigrisea:* **(A,B)** from FHMU 1651; **(C)** from MHKMU L.P. Tang 3251, holotype.

**FIGURE 12:**
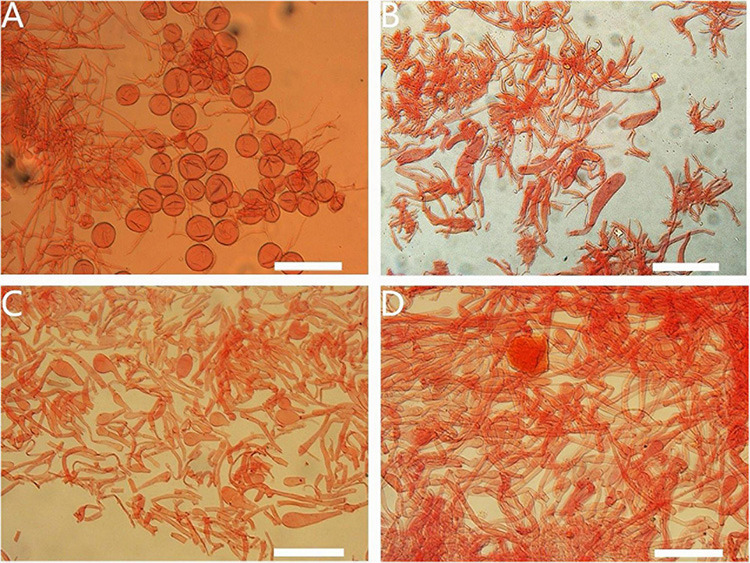
Annulus of *Amanita* sect. *Validae*: **(A)** *A. parvifritillaria* (MHKMU T. Huang 412); **(B)** *A. pseudofritillaria* (MHKMU T. Huang 398); **(C)** *A. parvigrisea* (MHKMU L.P. Tang 3251); **(D)** *A. yangii* (MHKMU M. Mu 660). *Bars:* **(A,B) =** 100 μm; **(C,D) =** 50 μm.

MycoBank no.: MB 844370 (Figures 4D, 5D, 6C, 11, 12C).

Holotype—China. Hainan Province, Baisha Li Autonomous County, Yinggeling Nature Reserve, 19°1′19″N, 109°23′48″E, elevation 580 m, 14 August 2020, *L.P. Tang 3251* (MHKMU L.P. Tang 3251); GenBank accessions: ON768716 (ITS), ON768741 (LSU).

Etymology—*parvigrisea*, from *parus* = small, and *grisea* = gray, referring to its small basidiomata and the color of the pileus.

Diagnosis—Similar to *A. innatifibrilla* but differs in its light gray pileus, subapical annulus, subglobose bulb, and subglobose basidiospores.

*Basidiomata* small. *Pileus* 3.4–3.8 cm in diam., convex to applanate, lacking an umbo or depression at the center, light gray to pearl gray, smoke gray (4C1, 5C1, 6C1) or gray yellowish to grayish beige (4C2–3), paler toward the margin, radiating fibrils; volval remnants on pileus patches to verrucose or subconical, dirty white, easily removed; margin non-striate, non-appendiculate, sometimes radially rimose; *context* 0.2 cm thick at the center, white (1A1), unchanging. *Lamellae* 0.4 cm in width, free, slightly crowded, 56–80 pieces of primaries with 1–2 shorter ones between each pair, white (1A1); lamellae attenuate. *Stipe* 6–6.5 cm long, 0.4–0.9 cm in diam., tapering upward, often light gray to pearl gray (4B1, 4C1), covered with light gray to pearl gray (4B1, 4C1) minute squamules; *basal bulb* 1.4 cm in diam., subglose, white (1A1), sometimes covered light gray (4B1, 4C1) floccose to cottony volval remnants; *context* white (1A1), stuffed in the center. *Annulus* small, subapical, submembranous, friable, white, (1A1). *Taste* no record. *Odor* indistinct.

*Basidiospores* [40/2/2] (7.5) 8.0–9.0 (9.5) × 7.0–8.5 (9.0) μm, Q = 1.05–1.2, Q_*m*_ = 1.10 ± 0.17, mostly subglobose, sometimes broadly ellipsoid, colorless, thin-walled, smooth, amyloid, apiculus small. *Basidia* 25–40 × 10–15 μm, clavate, 4-spored; sterigmata 4–5 μm long; basal septa lacking clamps. *Lamellar trama* bilateral. Mediostratum 20–45 μm wide, made up of abundant inflated cells 45–70 × 10–25 μm, subfusiform, ellipsoid to clavate; mixed with filamentous hyphae 2–7 μm in diam., abundant; vascular hyphae scarce. The lateral stratum is made up of abundant inflated cells 35–73 × 15–25 μm, subfusiform to ellipsoid; mixed with filamentous hyphae 3–7 μm in diam. *Subhymenium* 20–40 μm thick, with 2–3 layers of subglobose to ellipsoid or irregular cells, 9–20 × 6–15 μm. *Lamellar edge* is mainly composed of abundant inflated cells 23–35 × 20–32 μm, hyaline, numerous subglobose, ellipsoid, single or in short chains, mixed with scattered filamentous hyphae 2–7 μm in diam. Insufficient material of volva remnants on pileus to perform observing. *Pileipellis* 80–140 μm thick; upper layer 50–100 μm thick, non- or slightly gelatinized, composed of filamentous hyphae 2–5 μm in diam., subradially to somewhat interwoven, thin-walled, colorless or yellowish; lower layer 30–40 μm thick, composed of radially and compactly arranged filamentous hyphae 3–10 μm in diam., colorless or yellowish; vascular hyphae scarce. *Stipe trama* is primarily composed of longitudinally arranged and abundant terminal cells 50–130 × 10–35 μm, clavate to long clavate; mixed with scattered to more filamentous hyphae 2–8 μm in diam.; vascular hyphae scarce. *Annulus* composed of radially arranged elements: very abundant to dominant filamentous hyphae 1–7 μm in diam., colorless, thin-walled; scarce inflated cells 15–30 × 7–25 μm, subglobose, subfusiform to ellipsoid, colorless, thin-walled; vascular hyphae scarce. *Clamps* are absent in all parts of basidiomata.

Habitat—Solitary or scattered on soil in a tropical broad-leaved forest; in summer.

Distribution—Hainan Province, China.

Additional specimen examined—China. Hainan Province, Baisha Li Autonomous County, Yinggeling Nature Reserve, 19°2′57″ N, 109°33′46″ E, elevation 850 m, 4 August 2015, *N.K. Zeng 2538* (MHKMU N.K. Zeng 2538).

Notes—*Amanita parvigrisea* is distinguished by its small basidiomata, a gray pileus with radiating fibrils and patches remnants, a light gray stipe with a subapical white annulus and a bulbous base, and subglobose basidiospores.

Morphologically, there are two species of sect. *Validae* from the Chinese tropics to subtropics, which resemble *A. parvigrisea* in the size and color of the pileus. *Amanita innatifibrilla* Zhu L. Yang ex Zhu L. Yang, Yang-Yang Cui, & Qing Cai, also originally from Hainan, is distinctive by slightly darker volval remnants on gray pileus, smaller and ventricose basal bulb (0.6–1 cm in diam.), median annulus, and slightly smaller and wider basidiospores (8.0–9.0 × 7.0–8.5 μm, Q_*m*_ = 1.18) (Cui et al., 2018). *Amanita parvifritillaria* originally from Yunnan has a slightly paler pileus, paler volval remnants on pileus, whitish stipe almost glabrous or covered with indistinct squamules, basal bulb with incompletely concentric rings, and slightly wider basidiospores (Cui et al., 2018).

Another species from tropical South American Guyana, *A. guyanensis*, is similar to the new taxon. However, the former differs in its bigger and dark gray-brown pileus (up to nearly 10 cm), white stipe with angled basal bulb, and slightly bigger basidiospores (Mighell et al., 2019).

Interestingly, *A. parvigrisea* did not cluster with those mentioned taxa in phylogeny. Phylogenetically, this new taxon was rather isolated in the sect. *Validae*, and current data did not suggest any species closely related to this new species.

**FIGURE 13:**
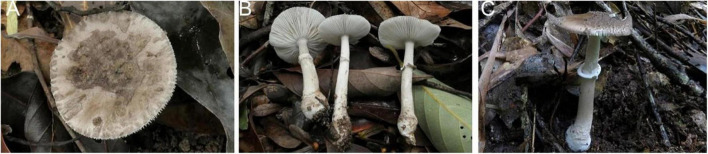
Basidiomata of *Amanita parvifritillaria*: **(A,B)** from MHKMU T. Huang 412; **(C)** from MHKMU N.K. Zeng 3596.

*Amanita parvifritillaria* Y.Y. Cui, Q. Cai & Zhu L. Yang, Fungal Diversity 91: 199 (2018) (Figures 4E, 5E, 6D, 7D, 12A, 13).

The following description is based on our specimens.

*Basidiomata* small. *Pileus* 3–4 cm in diam., grayish brown; gray *volval remnants on pileus* patches, verrucose to conical; margin non-striate or occasionally with short striations, non-appendiculate; *context* white, unchanging. *Lamellae* 0.5 cm in width, free, crowded, about 55–80 primaries with 1–2 shorter ones between each pair, white; lamellae attenuate. *Stipe* 5.7–6.5 cm long, 0.4–0.5 cm in diam., whitish, glabrous to covered with grayish fibrous squamules; *basal bulb* 0.8–1.1 cm in diam., slightly expanded or subglobose, covered with grayish floccose volval remnants, sometimes arranged irregularly or in incompletely concentric rings; *context* white, unchanging. *Annulus is* subsuperior to the median, white (1A1), grayish on the edges. *Basal mycelium* white. *Taste* and *Odor* no records found.

*Basidiospores* [40/2/2] (6.0) 6.5–8.5 (9.0) × (5.5) 6.0–8.0 μm, Q = 1–1.16, Q_*m*_ = 1.08 ± 0.17, mostly subglobose, colorless, thin-walled, smooth, amyloid, apiculus small. *Basidia* 23–40 × 6–11 μm, clavate, 4-spored; sterigmata 4–5.5 μm long; basal septa lacking clamps. *Clamps* are absent in all parts of basidiomata.

Habitat—Solitary or scattered on soil in tropical broad-leaved forests; in summer.

Distribution—Hainan and Yunnan Province.

Additional specimens examined—China. Hainan Province, Baisha Li Autonomous County, Yinggeling Nature Reserve, 19°1′19″N, 109°23′48″E, elevation 529 m, 14 August 2020, *T. Huang 412* (MHKMU T. Huang 412). Yunnan Province, Jinping Watershed National Nature Reserve Wutai Mountain, 22°36′36″N, 102°31′36″E, elevation 1,200 m, 12 July 2018, *N.K. Zeng 3596* (MHKMU N.K. Zeng 3596).

Note—*Amanita parvifritillaria* is a recently described taxon, originally from Yunnan, China (Cui et al., 2018). In total, two collections from Hainan were clustered with the holotype from Yunnan without a genetic difference. This is the first time that *A. parvifritillaria* has been reported outside Yunnan Province. Based on new collections, this taxon should be summarized as follows: small basidiomata, grayish brown pileus with grayish patch remnants, margin non-striate or sometimes with short striations, non-appendiculate, whitish stipe covered grayish fibrous squamules, basal bulb covered grayish floccose remnants, and subglobose basidiospores. Compared with Hainan specimens, *A. parvifritillaria* has pyramidal to conical volval remnants and ellipsoid to fusiform basal bulb in Yunnan specimens.

*Amanita pseudofritillaria* L.P. Tang, T. Huang & N.K. Zeng sp. nov.

**FIGURE 14:**
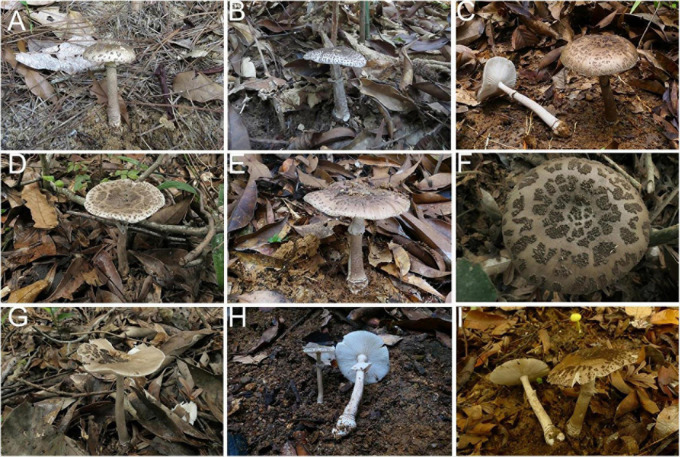
Basidiomata of *Amanita pseudofritillaria* and *A. fritillaria*: **(A–F)** *A. pseudofritillaria.* **(A)** from MHKMU N.K. Zeng 3433; **(B)** from MHKMU N.K. Zeng 3051; **(C)** from MHKMU N.K. Zeng 4270; **(D)** from MHKMU T. Huang 408; **(E)** from MHKMU N.K. Zeng 4268; **(F)** from MHKMU T. Huang 398, holotype. **(G–I)** *A. fritillaria*. **(G)** from MHKMU T. Huang 410; **(H)** from MHKMU N.K. Zeng 4437; **(I)** from MHKMU L.P. Tang 3253.

MycoBank no.: MB 844372 (Figures 4H, I, 5F, 6E, 7E, 12B, 14A–F).

Holotype—China. Hainan Province, Baisha Li Autonomous County, Yinggeling Nature Reserve, 19°1′19″N, 109°23′48″E, elevation 580 m, 14 August 2020, *T. Huang 398* (MHKMU T. Huang 398); GenBank accessions: ON768708 (ITS), OP056467 (*rpb2*).

Etymology—*pseudofritillaria*, from *pseudo*- = false-, and *fritillaria* = *Amanita fritillaria*, is proposed because this species is similar to *A. fritillaria*.

Diagnosis—Similar to *A. fritillaria* but differs in its darker color basidiomata, apical to the subapical annulus, slightly bigger bulb, and broadly ellipsoid basidiospores.

*Basidiomata* medium to large. *Pileus* up to 10 cm in diam., hemispherical at young, convex to applanate, sometimes slightly depressed at the center, putty or birch gray (3B2, 4D3, 4B2) to orange gray (5B2–3, 5C2–3, 5D2), paler toward margin; *volval remnants on pileus* verrucose to patches, mouse gray or neutral gray (6C1, 6D1) to blackish (6E1, 6F1); margin non-striate, non-appendiculate; *context* 0.3–1 cm thick at the center, white (1A1), unchanging. *Lamellae* 0.4–1.2 cm in width, free, crowded, about 100–140 primaries with 1–2 shorter ones between each pair, white (1A1); lamellae attenuate, plentiful. *Stipe* up to 15 cm long, 1.8 cm in diam., subcylindrical or slightly tapering upward, birch gray (3B2, 4D3) to orange gray, even blackish (3D2, 5C2, 6C2), covered with concolorous floccose squamules; *basal bulb* up to 2.5 cm in diam., subglobose, top-shaped to somewhat enlarged, upper part covered with gray (5C2) to gray blackish (6E1, 6F1) volval remnants, more or less arranged in one to several incompletely concentric rings; *context* white (1A1), stuffed in the center, sometimes turning slightly red brown when cutting or bruising; *Annulus* small, apical to subapical, membranous, white (1A1). *Taste* no record. *Odor* is a bit strong.

*Basidiospores* [80/4/2] 7.0–8.5 (9.0) × 5.5–7.0 (7.5) μm, Q = (1.09) 1.12–1.33 (1.36), Q_m_ = 1.26 ± 0.20, broadly ellipsoid to ellipsoid, colorless, thin-walled, smooth, amyloid, apiculus small. *Basidia* 29–33 × 10.5–12 μm, clavate, 4-spored; sterigmata 3–4 μm long; basal septa lacking clamps. *Lamellar trama* bilateral. Mediostratum 20–40 μm wide, composed of abundant inflated cells 30–90 × 13–27 μm, subfusiform, ellipsoid to clavate; mixed with filamentous hyphae 3–10 μm in diam.; vascular hyphae scarce. The lateral stratum is composed of abundant inflated cells 35–80 × 8–31 μm, subfusiform to ellipsoid; mixed with filamentous hyphae 3–10 μm in diam. *Subhymenium* 15–35 μm thick, with 2–3 layers of cells 10–23 × 8–20 μm, subglobose to ellipsoid or irregular. *Lamellar edge* is mainly composed of inflated cells 27–45 × 22–35 μm, hyaline, numerous pyriform, fusiform, single or in short chains, mixed with scattered filamentous hyphae 3–10 μm in diam. *Volval remnants on pileus* composed of more or less vertically arranged elements: very abundant inflated cells 20–83 × 11–73 μm, subglobose, subfusiform to ellipsoid, colorless to yellowish, thin-walled, terminal or in chains of 2–3; mixed with scarce to more filamentous hyphae 2–10 μm in diam., colorless or yellowish, thin-walled, branching; vascular hyphae scarce. *Volva remnants on stipe base* are similar to the structure of volva remnants on pileus, but with more filamentous hyphae. *Pileipellis* 30–60 μm thick; upper layer 15–40 μm thick, non- or slightly gelatinized, composed of filamentous hyphae 3–9 μm in diam., thin-walled, colorless or yellowish, subradially to somewhat interwoven; lower layer 15–30 μm thick, composed of filamentous hyphae 3–10 μm in diam., colorless or yellowish, radially and compactly arranged; vascular hyphae scarce. *Stipe trama* composed of longitudinally arranged elements: fairly abundant terminal cells 40–240 × 10–30 μm, clavate to long clavate; mixed with scattered to abundant filamentous hyphae 3–8 μm in diam.; vascular hyphae scarce. *Annulus* composed of radially arranged elements: very abundant to dominant filamentous hyphae 3–9 μm in diam., colorless, thin-walled; mixed with inflated cells scarce; vascular hyphae scarce. *Clamps* are absent in all parts of basidiomata.

Habitat—Solitary or scattered on soil in a tropical broad-leaved forest; in summer.

Distribution—Guizhou, Hainan, and Yunnan Province, China.

Additional specimens examined of *A. pseudofritillaria*—China. Guizhou Province, Sandu Aquarium Autonomous County, 26°4′15″N, 107°52′21″E, elevation 570 m, 17 August 2018, *M. Mu 69* (MHKMU M. Mu 69); Gulu Scenic Spot, 25°55′17″N, 107°52′16″E, elevation 445 m, 19 August 2018, *M. Mu 80* (MHKMU M. Mu 80). Hainan Province, Baisha Li Autonomous County, Yinggeling Nature Reserve, elevation 550 m, 4 July 2017, *N.K. Zeng 3051* (MHKMU N.K. Zeng 3051); *ibid.*,19°1′19″N, 109°23′48″E, elevation 529 m, 14 August 2020, *T. Huang 408* (MHKMU T. Huang 408); *ibid.*, elevation 650 m, 2 July 2020, *N.K. Zeng 4268* (MHKMU N.K. Zeng 4268); *ibid.*, elevation 670 m, 2 July 2020, *N.K. Zeng 4270* (MHKMU N.K. Zeng 4270); Ledong Li Autonomous County, Jianfengling National Forest Park, 18°44′33″N, 108°50′32″E, elevation 850 m, 27 June 2018, *N.K. Zeng 3433* (MHKMU N.K. Zeng 3433). Yunnan Province, Kunming, Xiaoshao township, 25°12′37″N, 102°55′54″E, elevation 2,143 m, 14 July 2015, *L.P. Tang 1871* (MHKMU L.P. Tang 1871); Wuding county, Gaoqiao, 25°36′51″N, 102°09′23″E, elevation 2,090 m, 20 August 2016, *L.P. Tang 2312* (MHKMU L.P. Tang 2312); Chuxiong Yi Autonomous Prefecture, Nanhua County, Zixi Mountain Forest Park, 25°04′46″N, 101°25′31″E, elevation 2013 m, 5 August 2020, *T. Huang 327* (MHKMU T. Huang 327); *ibid.*, elevation 2170 m, 4 August 2020, *M. Mu 607* (MHKMU M. Mu 607); *ibid.*, elevation 1971 m, 5 August 2020, *Y.J. Pu 290* (MHKMU Y.J. Pu 290); *ibid.*, elevation 1971 m, 5 August 2020, *Y.J. Pu 295* (MHKMU Y.J. Pu 295); *ibid.*, elevation 2224 m, 4 August 2020, *W.H. Zhang 340* (MHKMU W.H. Zhang 340); *ibid.*, elevation 2013 m, 5 August 2020, *W.H. Zhang 359* (MHKMU W.H. Zhang 359).

Specimens examined of *A. fritillaria*—China. Hainan Province, Baisha Li Autonomous County, Yinggeling Nature Reserve, 19°01′15″N, 109°25′42″E, elevation 700 m, 12 July 2020, *N.K. Zeng 4437* (MHKMU N.K. Zeng 4437); *ibid.*, elevation 529 m, 14 August 2020, *T. Huang 406* (MHKMU T. Huang 406); *ibid.*, elevation 529 m, 14 August 2020, *T. Huang 410* (MHKMU T. Huang 410); *ibid.*, elevation 580 m, 14 August 2020, *L.P. Tang 3253* (MHKMU L.P. Tang 3253); *ibid.*, elevation 722 m, 15 August 2020, *M. Mu 691* (MHKMU M. Mu 691).

Notes—*Amanita pseudofritillaria* is characterized by basidiomata medium to large; pileus brownish white to grayish brown; volval remnants on pileus verrucose to patches, yellowish brown to brown-gray; stipe orange-gray, covered with floccose, brownish gray to grayish black squamules; basal bulb concolorous, upper part covered with brownish gray to grayish black squamules, arranged in incompletely concentric rings; annulus membranous, apical to subapical, whitish above, gray below; basidiospores subglobose to broadly ellipsoid; flesh white, turning slightly red brown cutting or bruising.

Morphologically, there are several taxa, which are comparable to the new taxon. *Amanita fritillaria* (Figures 4G, 14G–I), originally from north India, differs in its slightly light color basidiomata, whitish stipe, almost median annulus, smaller slightly turbinate bulb (less than 1.5–2 cm in diam.), and smaller and rounder basidiospores (6.0–7.6 × 5.4–6.6, Q_m_ = 1.1–1.15) (Corner and Bas, 1962; Kumar et al., 1990).

*Amanita lacerosquamosa*, originally from Yunnan, China, is recognized by light grayish brown basidiomata, a median annulus, and globose to subglobose basidiospores (Zhang et al., 2020).

*Amanita parvifritillaria*, originally from Yunnan, China, is different in its small basidiomata, sometimes the pileus with short striae on the margin, paler volval remnants, median annulus, and subglobose to broadly ellipsoid basidiospores (Cui et al., 2018; our observation in this study).

*Amanita spissacea* from Japan has a more floccose stipe, large patches on the pileus, and powdery volval remnants on the base of the white stipe (Imai, 1933, 1938).

Phylogenetically, ITS data and the concatenated data of nLSU*-rpb2* strongly supported *A. pseudofritillaria* and *A. fritillaria* as the sister species. It is worth noting that the two close species cannot be identified by LSU sequences.

*Amanita pseudosculpta* L.P. Tang & T. Huang, sp. nov.

MycoBank no.: MB 844373 (Figures 4B, 5B, 6B, 7B, 8B, 9).

Holotype—China. Hainan Province, Ledong Li Autonomous County, Jianfengling National Forest Park, in a tropical broad-leaved forest, 18°44′33″N, 108°50′32″E, elevation 1,000 m, 10 August 2020, *L.P. Tang 3167* (MHKMU L.P. Tang 3167); GenBank accessions: no data (ITS), ON768735 (LSU), OP056472 (*rpb2*).

Etymology—*pseudosculpta*, from *pseudo*- = false-, and *sculpta* = *Amanita sculpta*, because this species is similar to *A. sculpta*.

Diagnosis—Similar to *A. sculpta* but differs in its smaller basidiomata, yellowish brown pileus, and ovoid to subglose basal bulb almost smooth without any warts.

*Basidiomata* very large. *Pileus* 16–18 cm in diam., convex at young, then applanate, slightly exceeding gills, camel brown, yellowish brown (6D4–5) to cinnamon brown at young, slightly darker when mature; volval remnants on pileus warts 1 cm in height and 0.8 cm in width at the center, becoming smaller toward the margin, pyramidal, distant, not felted even at bud stage, cacao brown or leather brown (6E5–6), attached by a pale or dingy white, radiating base; margin non-striate, appendiculate with ragged partial veil, dingy white (–A1), somewhat brownish to linoleum brown (5E6–7) when bruising or old. *Context* 1 cm thick at the center, dingy pinkish to pink (9A2–3, 9B2–3) when young, liver brown to oxide red (8E8, 8F8) when mature or bruising. *Lamellae* 1.5–1.9 cm in width, nearly free, crowd, 150–185 pieces of primaries with 1 shorter one between each pair, pinkish white, pale pink to shell pink (7A2, 8A2) at young, camel brown (6D4–5) to liver brown or dark brown (8F5–6) when bruising, often with a dingy white edge from remnants of the partial veil; lamellae attenuate. *Stipe* 20–25 cm long, 2–3 cm in diam., golden brown to linoleum brown (5D7–8, 5E6–7), covered with floccose to cottony volval remnants at above; *basal bulb* 4–6 cm in diam., ovoid to subglose, almost smooth, sometimes covered with a few of floccose to cottony volval remnants; *context* same to the context of the pileus, pinkish at young, liver brown to oxide red when bruising. *Annulus* absent, poorly developed in shape even at the bud, fairly friable, floccose to cottony, dingy white (–A1), leaving at the edge of the pileus, at edges of lamellae, and on the stipe. *Taste and odor* no records found.

*Basidiospores* [40/2/2] 8.0–10.5 × 8.0–10.5 μm, Q = 1–1.06, Q_m_ = 1.02 ± 0.02, globose, colorless, thin-walled, smooth, amyloid, apiculus small. *Basidia* 48–60 × 12–18 μm, clavate, 4-spored; sterigmata 3–5 μm long; basal septa lacking clamps. *Lamellar trama* bilateral. Mediostratum 25–40 μm wide, composed of abundant inflated cells 38–90 × 10–28 μm, subfusiform, ellipsoid to clavate; mixed with filamentous hyphae 2–10 μm in diam.; vascular hyphae scarce. The lateral stratum is composed of abundant inflated cells 25–66 × 8–12 μm, subfusiform to ellipsoid; mixed with filamentous hyphae 2–8 μm in diam. *Subhymenium* 20–35 μm thick, with 2–3 layers of cells 7–24 × 6–10 μm, subglobose to ellipsoid or irregular. *Lamellar edge* is mainly composed of numerous inflated cells 26–52 × 20–30 μm, hyaline, pyriform, globose, single or in short chains, mixed with more filamentous hyphae 2–8 μm in diam. *Marginal cells* are dominantly composed of inflated cells 105–230 × 20–40 μm, subfusiform to fusiform, mixed with more filamentous hyphae 3–12 μm in diam. *Volval remnants on pileus* composed of more or less vertically arranged elements: very abundant inflated cells 32–73 × 15–62 μm, subglobose, subfusiform to ellipsoid, colorless to yellowish, thin-walled, terminal or in chains of 2–3; mixed with scattered to a few filamentous hyphae 3–10 μm in diam., colorless or yellowish, thin-walled, branching; vascular hyphae scarce. *Volval remnants on stipe* and *stipe base* are similar to the structure of volval remnants on pileus, but with less filamentous hyphae. *Pileipellis* 40–100 μm thick; upper layer 20–60 μm thick, non- or slightly gelatinized, composed of subradially to somewhat interwoven filamentous hyphae 3–10 μm in diam., thin-walled, colorless or yellowish; lower layer 20–50 μm thick, composed of radially and compactly arranged filamentous hyphae 3–14 μm diam., colorless or yellowish; vascular hyphae scarce. *Stipe trama* is composed of longitudinally arranged elements: numerous terminal cells 120–365 × 26–55 μm, clavate to long clavate; scattered to more filamentous hyphae 5–10 μm in diam.; vascular hyphae scarce. *Clamps* are absent in all parts of basidiomata.

Habitat—Solitary or scattered on soil in a tropical broad-leaved forest; in summer.

Distribution—Hainan Province, China.

Additional specimens examined—China. Hainan Province, Ledong Li Autonomous County, Jianfengling National Forest Park, a tropical broad-leaved forest, 18°44′33″N, 108°50′32″E, elevation 1000 m, 10 August 2020, *T. Huang 342* (MHKMU T. Huang 342); *ibid*., elevation 1000 m, 10 August 2020, *W.H. Zhang 379* (MHKMU W.H. Zhang 379).

Notes—*Amanita pseudosculpta* is distinguished by very large and yellow brown basidiomata, large and distant warts on pileus which come off easily, subglobose basal bulb, and globose basidiospores. Morphologically, the new taxon strongly resembles *A. cacaina*, *A. sculpta*, and *A. westii*. In China, this new species and another one *A. cacaina* were likely to be mistaken for *A. sculpta* (Yang, 1997, 2005, 2015; Tang, 2015; Cui et al., 2018; Zeng and Jiang, 2020). For comparisons, see the note of *A. cacaina*. In the phylogenetic trees, *A. pseudosculpta* was sister to *A. sculpta* with strong support.

*Amanita yangii* L.P. Tang & T. Huang sp. nov.

**FIGURE 15:**
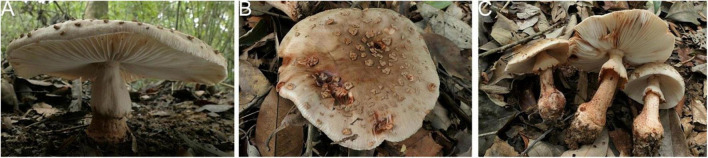
Basidiomata of *Amanita yangii*: **(A)** from MHKMU M. Mu 660, holotype; **(B,C)** from MHKMU X. Na 100.

MycoBank no.: MB 844371 (Figures 4F, 5G, 6F, 7F, 12D, 15).

Holotype—China. Hainan Province, Baisha Li Autonomous County, Yinggeling Nature Reserve, 19°1′19″N, 109°23′48″E, elevation 530 m, 13 August 2020, *M. Mu 660* (MHKMU M. Mu 660); GenBank accessions: ON768731 (ITS), ON768731 (LSU); OP056473 (*rpb2*).

Etymology— *yangii*, named in honor of Dr. Zhu L. Yang, a famous Chinese mycologist, for his important contribution to the fungal taxonomy, especially to the study of *Amanita*.

Diagnosis—Similar to *A. orsonii* but differs in its larger and more robust basidiomata, a gray white pileus, an apical and thick annulus, and globose to subglobose basidiospores.

*Basidiomata* large. *Pileus* 9–14 cm in diam., rounder when young, convex to applanate, lacking an umbo or depression at the center, whitish to gray white (2B2, 2B3), with light gray to grayish, mouse gray (5C4, 5D4, 5E4) tinge at center or maturity, becoming reddish to red brown, cacao brown, leather brown (6E6, 6E7) when injured; *volval remnants (warts) on pileus* usually flattened patches to felted at a young stage, almost becoming small pyramidal, conical, subconical to verrucose at mature, gray-white (2B2, 2B3), usually with reddish or brick red apices when bruising; non-appendiculate and non-striate margin at first, then becoming faintly striate at maturity. *Context* 0.6 cm thick in the center of the pileus, white (1A1), reddish brown to brick red, leather brown (6E6, 6E7) when injured. *Lamellae* 0.8 cm in width, 100–120 pieces of primaries with 0–3 shorter ones between each pair, free, crowded, cream to white (1A1), reddish brown to brick red (6E6, 6E7) when injured; lamellae attenuate, plentiful. *Stipe* 7–8 cm long, 1–2.5 cm in diam., cylindrical, cream to whitish, often reddish brown to brick red, leather brown (6E6, 6E7) when bruising, glabrous or covered with minute squamules; *basal bulb* 2.5–3.2 cm in diam., subglobose, concolorous with stipe; *context* white (1A1), stuffed in the center. *Annulus* large, apical, persistent, thick, skirt-shaped, upper surface with distinct striations, adorned with cottony remnants at the edge, cream to white (1A1) on both surfaces, sometimes with reddish tints when injured. *Taste and Odor* are indistinct.

*Basidiospores* [40/2/2] (6.0) 6.5–8.0 (8.5) × (5.0) 6–7 (7.5) μm, Q = 1.03–1.15 (1.23), Q_m_ = 1.08 ± 0.17, globose to subglobose, colorless, thin-walled, smooth, amyloid; apiculus small. *Basidia* 32–43 × 10–11 μm, clavate, 4-spored; sterigmata 3–5 μm long; basal septa lacking clamps. *Lamellar trama* bilateral. Mediostratum 30–70 μm wide, composed of abundant inflated cells 30–100 × 11–20 μm, subfusiform, ellipsoid to clavate; mixed with filamentous hyphae 3–11 μm in diam.; vascular hyphae scarce. The lateral stratum is composed of abundant inflated cells 40–103 × 10–16 μm, subfusiform to ellipsoid; mixed with filamentous hyphae 3–10 μm in diam. *Subhymenium* 20–50 μm thick, with 2–3 layers of inflated cells, 12–30 × 5–18 μm, subglobose to ellipsoid or irregular. *The lamellar edge* is primarily composed of inflated cells 14–30 × 9–12 μm, numerous globose to subglobose, pyriform, single or in short chains; mixed with scattered filamentous hyphae 3–10 μm in diam. *Volval remnants on pileus* dominated by inflated cells 25–156 × 15–66 μm, subglobose, subfusiform to ellipsoid, colorless to yellowish, thin-walled, terminal or in chains of 2–3; mixed with a few filamentous hyphae 3–13 μm in diam., colorless or yellowish, thin-walled, branching; vascular hyphae scarce. *Pileipellis* 60–125 μm thick; upper layer 30–80 μm thick, non- or slightly gelatinized, composed of subradially to somewhat interwoven filamentous hyphae 2–9 μm in diam., thin-walled, colorless or yellowish; lower layer 10–45 μm thick, composed of radially and compactly arranged filamentous hyphae 3–13 μm in diam., colorless or yellowish; vascular hyphae scarce. *Stipe trama* composed of longitudinally arranged elements: fairly abundant terminal cells 25–160 × 11–31 μm, clavate to long clavate; mixed with scattered filamentous hyphae 2–13 μm in diam.; vascular hyphae scarce. *Annulus* primarily composed of abundant filamentous hyphae 2–10 μm in diam., colorless, thin-walled, inflated cells and vascular hyphae scarce; annular edge composed of very abundant inflated cells 35–70 × 10–20 μm, fusiform to clavate, colorless or yellowish, thin-walled; mixed with a few filamentous hyphae 2–7 μm in diam., colorless or yellowish, thin-walled; Clamps are absent in all parts of basidiomata.

Habitat—Solitary or scattered on soil in tropical broad-leaved forests; in summer.

Distribution—Hainan Province, China.

Additional specimen examined—China. Hainan Province, Baisha Li Autonomous County, Yinggeling Nature Reserve, 19°1′19″N, 109°23′48″E, elevation 580 m, 13 August 2020, *X. Na 100* (MHKMU X. Na 100).

Notes—*Amanita yangii* is in the field recognized by its large and robust basidiomata, gray white pileus, white stipe covered floccose scales, subglobose basal bulb (2.5–3.2 cm in diam.), an apical cream annulus, globose to subglobose basidiospores, and all parts of basidiomata reddish when injured.

In the section *Validae*, there are several species similar to their basidiomata having reddish tones when bruised, but they are quite different in the color of the pileus, the position and color of annulus, shapes, and sizes of basidiospores, and distributions.

*Amanita brunneolocularis* Tulloss, Ovrebo and Halling, originally described from Colombia, has a sordid flesh-color to somewhat reddish-brown pileus with a non-striate margin, the annulus with a gray lower surface, broadly ellipsoid to ellipsoid basidiospores, and discoloring reddish to brownish, ever nearly black (Tulloss et al., 1992).

*Amanita citrinoannulata* Y.Y. Cui, Q. Cai and Zhu L. Yang has a smaller and slender basidioma, a yellow-brown pileus with olivaceous tinge, yellow squamules on the stipe, broadly ellipsoid to ellipsoid basidiospores, and occurring in subtropical to temperate forests (Cui et al., 2018).

*Amanita flavorubescens* G.F. Atk. originally from North America is distinct in its bright yellow basidiomata, ovate bulb, basidiospores oboval, and the tendency to bruise slowly reddish to reddish brown (Atkinson, 1902; Jenkins, 1982; Kuo, 2013a).

*Amanita novinupta* Tulloss and J. Lindgr. originally described from USA. is distinct from *A. yangii* in its entirely white pileus at first, ellipsoid to subnapiform basal bulb, ellipsoid basidiospores, present clamps, and all part of basidiomata discolored pink to wine to red-brown when injured (Tulloss and Lindgren, 1994; Kuo, 2013b).

*Amanita orsonii* Ash. Kumar and T. N. Lakh., originally from north India, is different in its grayish red to reddish brown pileus; volva remnant as floccose, flat, polygonal scales and warts on pileus white at young, grayish brown at maturity; subapical to the medium annulus; and broadly ellipsoid to ellipsoid basidiospores (Tulloss et al., 2001; Cui et al., 2018).

*Amanita rubscens*, originally from Europe, is distinctive by brown pileus; the apex of stipe covered with fibrillose scales; basal bulb with pulverescent gray volval remnant; white annulus, unchanging; and ellipsoid basidiospores (Persoon, 1797; Yang, 2005, 2015).

Interestingly, *A. yangii* did not cluster with those mentioned taxa in phylogeny while LSU*-rpb2* data indicated that the new taxon was close to *A. guyanensis*. *Amanita guyanensis* from Guyana is recognized by its smaller basidiomata (pileus less than 10 cm in diam.); darker pileus (gray-brown); white stipe covered with gray floccose scales; smaller subglobose bulb (2–3 cm in diam.); and an annulus with a gray margin (Mighell et al., 2019).

## Discussion

The characters of sect. *Validae* were summarized by several different mycologists (Corner and Bas, 1962; Bas, 1969; Yang, 2005, 2015; Cui et al., 2018). Corner and Bas (1962) argued that the species in this section should have the following characters: pileus not exceeding gills, never appendiculate; remnants of volva on pileus easily washed off; gills white to cream; ring membranous; stipe with comparatively small bulb; and basidiospores globose to ellipsoid. The appendiculate edge of the pileus is the key character to distinguish section *Lepidella* (including most taxa of sect. *Roanokenses* in Cui et al. (2018) from sect *Validae*.

In this study, two new species (*A. cacaina* and *A. pseudosculpta*) have obviously appendiculate pileus and have no well-developed annulus even at the bud period. Their characters closely match the diagnosis of the sect. *Roanokenses* (Cui et al., 2018), but our multiple phylogenetic data support that the two species are members of the sect. *Validae*. Another taxon, *A. sculpta* which also has the appendiculate edge of the cap, was treated as a member of the sect. *Roanokenses* (Cui et al., 2018), but the molecular trees supported this species also belong to sect. *Validae*.

According to our observation, the shape of the stipe bulb and the shape of basidiospores seem to be useful to distinguish the two sections. Based on the new data, the diagnostic characters of the sect. *Validae* should be updated as follows: pileus white, cream to dark colors, even chocolate brown, sometimes pileus exceeding gills; pileus margin non-striate, occasionally with short striations, and mostly non-appendiculate, occasionally appendiculate; stipe bulb usually globose to subglobose, rare clavate; annulus present to absent, usually well-shaped, membranous; volva remnants often as verrucae, warts, floccose or patches, occasionally as short limb; basidiospores globose, subglobose to broadly ellipsoid (usually Q_m_ < 1.3_)_, amyloid; clamps absent; usually smell indistinct.

Undoubtedly, molecular evidence is necessary to understand the true range of some so-called “cosmopolitan” species. The discovery of these mentioned taxa that have an appendiculate pileus in sect. *Validae* poses a challenge to the definition of sect. *Roanokenses* and sect. *Validae*. These taxa, which have fusiform to ventricose or limbate bulb, broad ellipsoid to bacilliform basidiospores (usually Q_m_ > 1.3), strong odor, and clamped basidia, should be placed in sect. *Roanokenses*.

## Disclosure

All the experiments undertaken in this study comply with the current laws of the People’s Republic of China.

## Data availability statement

The datasets presented in this study can be found in online repositories. The names of the repository/repositories and accession number(s) can be found in this article/supplementary material.

## Author contributions

TH, L-PT, N-KZ, W-HZ, H-YH, SL, S-SL, BT, SJ, L-JS, and JM performed by material preparation, data collection, and analyses. The first draft of the manuscript was written by TH and L-PT, and other authors commented on the previous versions of the manuscript. All authors contributed to the study’s conception and design and read and approved the final manuscript.

## Footnotes

1
www.indexfungorum.org

2
https://www.ncbi.nlm.nih.gov
